# Density‐habitat relationships of white‐tailed deer (*Odocoileus virginianus*) in Finland

**DOI:** 10.1002/ece3.9711

**Published:** 2023-01-10

**Authors:** Jenni Poutanen, Angela K. Fuller, Jyrki Pusenius, J. Andrew Royle, Mikael Wikström, Jon E. Brommer

**Affiliations:** ^1^ Department of Biology University Hill, University of Turku Turku Finland; ^2^ Natural Resources Institute Finland Turku Finland; ^3^ Department of Natural Resources and the Environment, U.S. Geological Survey, New York Cooperative Fish and Wildlife Research Unit Cornell University Ithaca New York USA; ^4^ Natural Resources Institute Finland Joensuu Finland; ^5^ U.S. Geological Survey Eastern Ecological Science Center Laurel Maryland USA; ^6^ Finnish Wildlife Agency Helsinki Finland; ^7^ NOVIA University of Applied Sciences Ekenäs Finland

**Keywords:** non‐invasive genetics, *Odocoileus virginianus*, population density, spatial capture‐recapture, white‐tailed deer, wildlife ecology

## Abstract

In heterogeneous landscapes, resource selection constitutes a crucial link between landscape and population‐level processes such as density. We conducted a non‐invasive genetic study of white‐tailed deer in southern Finland in 2016 and 2017 using fecal DNA samples to understand factors influencing white‐tailed deer density and space use in late summer prior to the hunting season. We estimated deer density as a function of landcover types using a spatial capture‐recapture (SCR) model with individual identities established using microsatellite markers. The study revealed second‐order habitat selection with highest deer densities in fields and mixed forest, and third‐order habitat selection (detection probability) for transitional woodlands (clear‐cuts) and closeness to fields. Including landscape heterogeneity improved model fit and increased inferred total density compared with models assuming a homogenous landscape. Our findings underline the importance of including habitat covariates when estimating density and exemplifies that resource selection can be studied using non‐invasive methods.

## Graphical abstract

In heterogeneous landscapes, resource selection constitutes a crucial link between landscape and population‐level processes such as density. We studied factors influencing white‐tailed deer density and space use in Finland by using non‐invasively collected fecal DNA samples and Spatial Capture‐Recapture modeling. Here, we underlined the importance of incorporating habitat covariates when estimating density and exemplify that resource selection can be studied using non‐invasive methods.

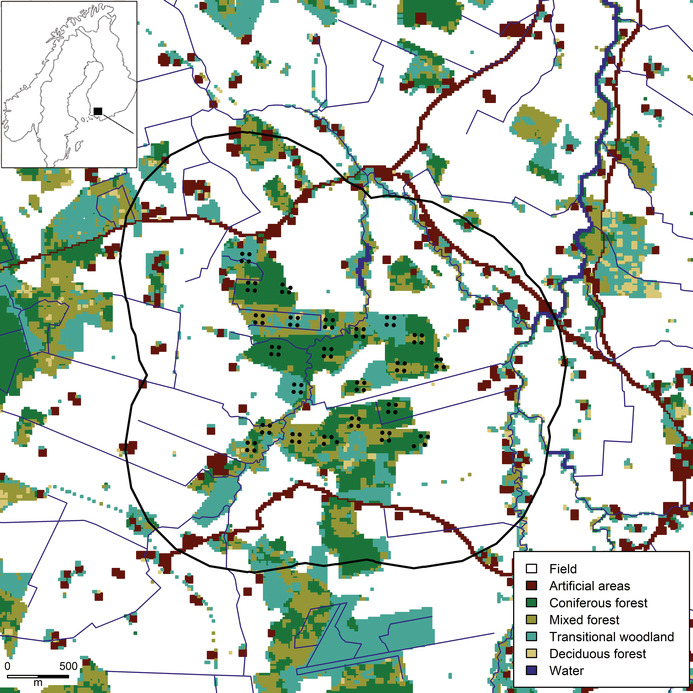

## 1 INTRODUCTION

Management of wild animal populations relies on understanding population abundance. Population densities are often dependent on habitat quality as animals rarely use all available habitats equally (Bjørneraas et al., 2012; Fretwell, 1969; Maier et al., 2005). Instead, animals preferentially select some habitats and avoid others, often resulting in higher densities of individuals in favored areas. Thus, understanding the resource selection of animals is an important part of management and conservation of many species as the actions for managing or maintaining the population differs among landscapes (Allen & Singh, 2016).

The preference of animals for certain habitats, i.e. the habitat (or resource) selection can be viewed as a hierarchical process with multiple orders (Johnson, 1980). Within a population, of interest is how individuals are distributed in relation to environmental features i.e. the location of home ranges of individuals (second‐order habitat selection), as well as within home‐range selection of habitats by individuals (third order). Commonly, habitat selection has been studied invasively using telemetry e.g. by attaching GPS or VHF collars on animals (e.g. Bose et al., 2018; Morris et al., 2016). Live capturing a large number of individuals, especially of large species, not only causes safety risks for animals and people but is also expensive. Therefore, even though information on space usage using telemetry can be detailed, it is often derived from information on only a limited number of individuals that may not be representative of the population. Thus, telemetry data often concerns individual‐level habitat selection rather than population‐level habitat selection. Non‐invasive genetic methods allow for the possibility to study animal resource use without physically marking and recapturing individuals, for instance by collecting feces (e.g. Granroth‐Wilding et al., 2017; Hagemann et al., 2018) or hair (e.g. O'Meara et al., 2018; Sun et al., 2017; Waits & Paetkau, 2005). Individual identification is obtained by extracting DNA from the samples and genotyping it to a sufficient resolution to allow obtaining unique individual‐specific genotypes. Spatial information of the individuals is then recorded from the sample (often termed capture) locations, which together provides spatially explicit records of individuals. The resulting genotypes and spatial encounter data can provide valuable population‐level information about space use (Fuller et al., 2016; Karen‐Giselle et al., 2022; Lindsø et al., 2022).

Spatially explicit records of individuals can be analyzed using spatial capture‐recapture (SCR) models, a spatial extension of long‐established capture‐recapture methods, in order to estimate population density (Efford, 2004; Royle et al., 2014). Apart from inferring density, SCR can also be simultaneously used to examine spatial distribution of individuals in the populations e.g. habitat selection (Royle, Chandler, Sun, & Fuller, 2013) and landscape connectivity (Fuller et al., 2016; Royle, Chandler, Gazenski, & Graves, 2013; Sutherland et al., 2015). SCR connects population‐level information to landscape structure by accounting for the location of the sampling sites and spatial variation in encounter probability due to habitat selection (Royle, Chandler, Sun, & Fuller, 2013). Because the SCR approach includes space explicitly, it allows inclusion of habitat covariates into the models of both density and detection probability. Second‐order habitat selection, i.e., locations of individuals on the landscape and their relation to environmental features, is modeled by SCR using the activity centers of individuals (i.e., where the probability to detect an individual is highest, e.g. home range centers) as a function of habitat covariates. To study the habitat use of individuals within their home ranges, i.e. third order habitat selection, the habitat structure around the sampling locations or traps can be incorporated into SCR analyses to model how covariates affect encounter probabilities (Royle et al., 2018). SCR can estimate the effect of certain habitat types on density and encounter probability, even if individuals are not directly encountered in that habitat, by predicting the locations of individual home range centers in the vicinity of the sample units. Non‐invasive DNA sampling with SCR has been previously used to study the relationship of population density and habitat structure (e.g. Berl et al., 2018; Brazeal et al., 2017; Gogoi et al., 2020; Lamb et al., 2018; Proffitt et al., 2015).

White‐tailed deer (*Odocoileus virginianus*) inhabit a large variety of terrestrial habitats from forest to savanna (Halls, 1984; Massé & Côté, 2009; Urbanek et al., 2012), and feed on various vegetation types from leaves and bark of trees to forbs, fruits and agricultural crops (Halls, 1984; Johnson et al., 1995; Weckerly & Nelson, 1990). In Finland, it has become one of the most important game species after its remarkable and continuing growth in abundance since 1934, when the species was introduced from North America (Kekkonen et al., 2012; Poutanen et al., 2022). The winter population size in 2021–2022 is estimated to be around 109,000 individuals (Aikio & Pusenius, 2022). The species is managed by hunting about half of its population size annually. It is listed as a non‐invasive alien species in Finland's National Strategy on Invasive Alien Species (Finnish Ministry of Agriculture and Forestry, 2012). One of the biggest impacts of white‐tailed deer on humans are deer‐vehicle collisions, but in the areas of the densest population the species can also cause damage to agriculture and forestry for instance by eating vegetable crops and tree seedlings. For the management of this species and defining hunting license quotas, it is important not only to estimate abundance but also to understand what habitat types the white‐tailed deer prefers in Finland. However, the Finnish white‐tailed deer population has not been thoroughly studied in terms of demography and habitat selection (Poutanen et al., 2022). To this end, we conducted a SCR based study using fecal DNA in southwestern Finland with the aim to understand how white‐tailed deer use available habitat types in a short time period (about 2–3 weeks) just prior to the start of the hunting seasons in 2016 and 2017. We expected white‐tailed deer densities to be highest in planted crop areas and young woodlands as these areas contain the most resources.

## 2 MATERIALS AND METHODS

### 2.1 Study area and sampling

We sampled fecal pellets of white‐tailed deer in 2016 and 2017. The study area was approximately 3 km^2^ in size and was located in southwestern Finland (central coordinate: 60° 51′ 56″ N, 22° 49′ 26″ E (WGS84)). The study area was covered by forest, and surrounded by agricultural fields, which is a typical landscape for the region. Forests in the study area had tree species composition typical to Finnish boreal forests, where the dominant tree species are Scots pine (*Pinus sylvestris*) and Norway spruce (*Picea abies*) while the most common deciduous trees were birches (*Betula* spp), European aspen (*Populus tremula*) and willows (*Salix* spp). Agricultural fields in the study area were used to grow seed crops (wheat, oat, rye). Climatically, this region is characterized by relatively mild winters (−7 to −1°C) and summers (10–23°C). Cervids apart from white‐tailed deer that occur in this area include European roe deer (*Capreolus capreolus*) and moose (*Alces alces*).

**FIGURE 1:**
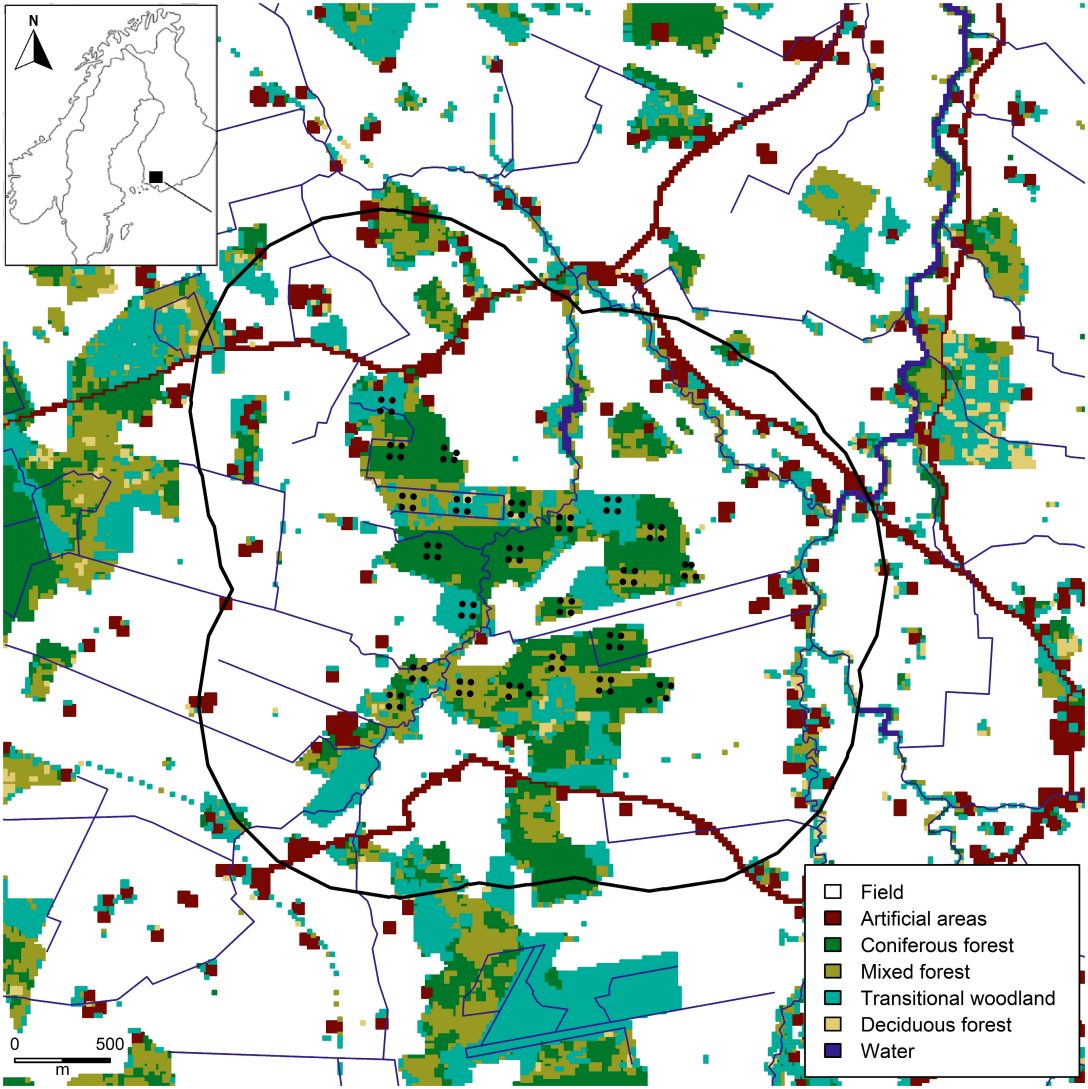
Landscape of the study area in Loimaa, Southwest Finland. Black dots indicate DNA sampling plots for white‐tailed deer. A black solid line represents the outline of the state space, which is defined as 1000 m buffer around the traps.

Sampling followed the protocol of Poutanen et al. (2019), but with small changes to the sampling design. We used a cluster design (Sun et al., 2014) with 23 clusters each including 4 sampling plots resulting in 92 sampled plots in total (Figure 1). Each plot was 20 m × 20 m in size and marked in the field with ribbons. The four plots forming a cluster were placed in a square with distances between the center coordinate of the four plots of approximately 60 m. The distances between the center of the clusters was approximately 300 m.

In order to sample a closed population without emigration, immigration, births or deaths, during a period when habitat such as planted fields are relatively stable, we conducted sampling in autumn before the white‐tailed deer hunting season, when migration is also limited and fawns of the year remain mostly with their mothers. In 2016, we sampled in September, but in 2017 sampling was initiated in August, due to the earlier timing of the hunting season. In 2016, we visited sampling plots weekly for three visits and in 2017 every 4 days for a total of six visits. During the first visit, the plots were cleared of deer pellets, which were then discarded and not used in the subsequent analysis. Because the first visit was this clearing visit, there were two sampling occasions in 2016 and five in 2017. In 2017, plots were visited more frequently than in 2016 because the results of the earlier study of the authors suggested that decreasing the sampling interval would allow collection of scats more frequently, reducing environmental exposure and DNA degradation, thus improving genotyping success rates (Poutanen et al., 2019). During each sampling occasion several fecal pellets were collected from each pellet group using a resealable plastic bag. All remaining pellets were removed to ensure accumulation of fresh pellets before the next sampling occasion. Samples were stored frozen at −20°C until genetic analysis could be conducted.

### 2.2 Genetic analysis

DNA extraction and individual identification followed the protocol of Poutanen et al. (2019). DNA was extracted using a commercially available DNA extraction kit (QIAamp DNA Stool Mini Kit, Qiagen, Valencia, California, USA). One extraction was done for each sample. We genotyped samples using 14 microsatellite markers and used the multitubes approach by performing three PCR replicates for each sample to minimize genotyping errors (Taberlet et al., 1996). We modified the microsatellite PCR protocol of Poutanen et al. (2019) by decreasing the final concentration of primer Rt5 from 0.2 μmol/L to 0.1 μmol/L and BSA concentration from 0.1 to 0.01 μg/μl. Samples which were successfully amplified with at least 11 loci were used to establish individual identity. Consensus genotypes were constructed with a rule that alleles of homozygous loci were needed to amplify three times and of heterozygous loci two times in order to be accepted as the final genotype (Jansson et al., 2014; Stansbury et al., 2014). When matching genotypes, we allowed two mismatches in different loci between the samples in order to accept them as representing the same individual. Thus, we required at least six matching loci between the samples to assign them to the same individual. The probability of identity (PI) values based on the six most uninformative loci were in the range of recommendations (PI = 0.0003 in 2016 and PI = 0.0001 in 2017, PID_sib_ = 0.02 in both years) (Waits et al., 2001). This suggests that the minimum of six matching loci between the samples was sufficient for individual identification. Consensus genotypes were matched to individuals using the software Cervus v. 3.0.7 (Kalinowski et al., 2007) and Gimlet v. 1.3.3 (Valière, 2002). At least one DNA sample of each identified individual were sexed with X‐ and Y‐ chromosome specific primer pair ZFX/ZFY following the protocol of Poutanen et al. (2019).

We used the consensus genotype data to calculate deviations from Hardy Weinberg Equilibrium using Genepop v. 4.2 (Rousset, 2008) and number of alleles and expected and observed heterozygosities using Gimlet (Valière, 2002). Fecal samples of white‐tailed deer can be confused with roe deer fecal pellets in the field. However, Poutanen et al. (2019) demonstrated that the panel of 12–14 microsatellite markers used in the PCR protocol allows discriminating between the DNA of these two species by amplifying properly only white‐tailed deer DNA. This microsatellite panel amplifies roe deer DNA only partly resulting in incomplete genotypes, which are discarded from subsequent analyses.

### 2.3 Spatial capture‐recapture density estimation

We estimated density using spatial capture‐recapture with the R package oSCR (Sutherland et al., 2016) in RStudio (RStudio Team, 2018). We used multi‐session SCR models with sampling year as a “session”. The main objective was to examine white‐tailed deer space use and how habitat structure is linked to density (*D*) and detection probabilities (*p*). First, we chose the top homogeneous model, i.e. the top model without habitat covariates, by Akaike Information Criterion (AIC), and then added habitat covariates to that model in the second step of model fitting. This simplified the model selection procedure by reducing possible combinations of covariates (Brazeal et al., 2017; Efford & Fewster, 2013). We chose the top homogeneous model among 42 different homogeneous models where we let *D*, *p* and *σ* (sigma, the parameter describing space use, defining rate at which detection probability declines as a function of distance from the sampling location (Royle et al., 2014)) vary by year, sampling occasion, and sex (we also fitted their interactions). When fitting heterogeneous models, *D* and *p* were let to vary by different habitat covariates. Second order resource selection is modeled by fitting habitat covariates to *D*, and third order selection by fitting habitat covariates to *p* (Royle et al., 2018). All heterogeneous models are modifications of the most supported homogeneous model. We also compared the overall predicted density estimates of the top homogeneous model with the top heterogeneous SCR model including habitat covariates. The state space was defined by a grid with resolution 120 m. A state space buffer of 1000 m around the traps was used, which is about 8 × *σ* based on the estimate of *σ* from this study (see Section 3) to ensure the buffer contain all home ranges of the sampled individuals.

Habitat covariates were defined using the open‐source Corine Land Cover data (European Union, Copernicus Land Monitoring Service 2012, European Environment Agency (EEA)). For water bodies, we used the vector data of waterways provided by National Land Survey of Finland (2018). We considered three different habitat covariates for density. First was a categorical habitat class variable with four different levels: agricultural fields, coniferous forests, mixed forests and transitional woodland/shrub. Other covariates on density included distance to artificial areas (e.g. buildings, roads and other artificially surfaced areas) and distance to water. These covariates were assigned to the state space by extracting them from the Corine Land Cover raster data with a function extr.rast() (oSCR package) using the habitat which was the most frequent when summarizing the Corine Land Cover raster values (resolution 20 m × 20 m) around the central coordinates of the state space pixel on the same resolution as the state space is defined (here 120 m). Artificial areas would have covered only 2% of the state space and we therefore ignored this habitat class. However, artificial areas and water bodies were included in the analysis by calculating the nearest distance from the state space pixel central coordinates to artificial area or water using R package rgeos (Bivand & Runde, 2021).

We included four different trap‐level covariates to study how landcover type affects capture probability (*p*). Differences in landcover of the sample plot may influence capture probability in various ways including the behavior of the animals. The first covariate was a categorical landcover class variable with three different levels: coniferous forest, mixed forests, transitional woodland/shrub (hereafter: transitional woodland). The other three covariates on capture probability were distance to agricultural areas, distance to artificial areas, and distance to water bodies. To define the landcover class for each trap location, the central coordinates of the sampling locations (“traps”) were buffered by 30 m (30 m buffer avoided overlap between buffers of adjacent traps when distance between the traps was 60 m) and the proportion of each landcover type was calculated for the buffered area. The landcover type with the largest proportion in the buffered area was assigned as the landcover class for that sampling location. If two or more landcover types existed in exactly the same proportions, then the class was defined using field notes on the landcover type of the central coordinate of the sampling location. Because deciduous forests were rare in the study area, this landcover class was ignored. To include agricultural areas, artificial areas and water bodies as covariates on capture probability, we calculated the nearest distance between the central coordinate of the sampling location and these landscape features using R package rgeos (Bivand & Runde, 2021).

For the SCR model, the state space of the study area was defined by buffering the minimum area rectangle of the sample units by 1000 m, producing a state‐space of 10.4 km^2^. Agricultural areas comprised 76% (548 of 725 pixels), coniferous forests 12% (88 pixels), mixed forests 6% (45 pixels) and transitional woodland 6% (44 pixels; Figure 1). Of the 92 sample plots, 51.1% (47 sample plots) were characterized as coniferous forest, 30.4% (28 plots) as mixed forest and 18.5% (17 plots) as transitional woodland.

## 3 RESULTS

### 3.1 Genetic analysis

We collected 300 white‐tailed deer fecal samples during two sampling occasions in 2016 and 401 samples during five sampling occasions in 2017 (Table 1). Samples were found on 72% (2016) and 79% (2017) of the 92 sample plots.

**TABLE 1:** Summary of the white‐tailed deer non‐invasive survey based on fecal samples collected on 92 sampling plots in 2016–2017 within our study area in Southwest Finland. Provided are the number of fecal samples and the number of these for which at least 11 microsatellite loci could be genotyped (allowing individual identification). The resulting number of individuals (of each sex) that were identified and their sex ratio. The total number of detections (of each sex) is broken down into how many individuals were detected once vs. multiple times (i.e. recaptured). The recapture rate is the fraction of individuals that were recaptured (detected more than once).

Year	2016	2017
No of collected samples	300	401
Successfully genotyped (≥11 loci)	94 (31%)	130 (32%)
No of individuals (♂/♀)	38 (12/26)	66 (25/41)
Sex ratio (♂/♀)	0.46	0.61
Total number of detections (♂/♀)	76 (16/60)	121 (40/81)
No of individuals detected once (♂/♀)	19 (10/9)	37 (15/22)
No of recaptured individuals (♂/♀)	19 (2/17)	29 (10/19)
Recaptured once (♂/♀)	5 (0/5)	11 (5/6)
Recaptured twice (♂/♀)	11 (2/9)	13 (5/8)
Recaptured three times (♂/♀)	1 (0/1)	4 (0/4)
Recaptured four times (♂/♀)	2 (0/2)	–
Recaptured six times (♂/♀)	–	1 (0/1)
Recapture rate	50%	44%

In total, 32% of the samples were successfully genotyped to the level permitting individual assignment (11 to 14 loci) in both years (Table 1). Genetic summary statistics of microsatellite loci are presented in Table A1. We identified 38 different white‐tailed deer individuals (26 females and 12 males) in 2016 and 66 individuals (41 females and 25 males) in 2017. In total 17 (14 females and 3 males) of the individuals captured in 2017 were also present in the 2016 data set. In 2016, samples of identified individuals were found on 43% of the plots and in 2017 in 51% of the plots. In 2016, we recaptured 50% of the individuals one to four times and in 2017, 44% of the individuals one to six times (Table 1). In 2016, only 17% of the genotyped males were recaptured, whereas 65% of the genotyped females were recaptured (Table 1). In 2017, 40% of the genotyped males and 46% of the females were recaptured.

### 3.2 White‐tailed deer density and space use

Among the set of homogeneous models evaluated (i.e. models without habitat covariates; Table A2), the top model supported that density differed between years and baseline detection probability varied between sampling occasions, sexes and years. The space use parameter *σ* varied with the interaction of sex and year (Table A2).

We included habitat covariates for density and detection probability in the top homogeneous model (i.e. model 1 in Table A2) and evaluated 24 different heterogeneous candidate models. According to the most supported heterogeneous model, white‐tailed deer density was dependent on landcover‐type (agricultural areas, coniferous forests, mixed forests and transitional woodland). The probability of detecting a white‐tailed deer varied with landcover‐type (coniferous forest, mixed forests, transitional woodland) and with distance to agricultural areas (Table 2; Table 3). Distance to water bodies and distance to artificial areas were not significant for either density or detection probability.

**TABLE 2:** Eight candidate spatial capture‐recapture models (1–8) for estimating density of white‐tailed deer by assuming a heterogeneous landscape compared with the null model assuming a homogeneous landscape (model 9). The null model includes session (year 2016, 2017) specific density (*D*), with capture probability (*p*) dependent on sampling occasion (*t*), sex (male, female) and session. The space use parameter sigma (sig) was both session and sex‐specific. The heterogeneous models (1–8) additionally include habitat covariates (habitatclass; agricultural areas, coniferous forest, mixed forest, transitional woodland) for density (*D*) and further allow detection probability (*p*) at an fDNA sampling plot to be a function of habitat class, distance to water (DistanceWater), distance to artificial surface (DistanceArtificial), and distance to agricultural area (DistanceAgr). The coefficients of detection of the most parsimonious model (model 1) are detailed in Table 3.

	Model	logL	*K*	AIC	dAIC	Weight	CumWt
1	*D*(session + habitatclass) *p*(*t* + sex + session + DistanceAgr + habitatclass) sig(session*sex)	799.3700	20	1638.740	0.000000	8.332334 e‐01	0.8332334
2	*D*(session + habitatclass) *p*(*t* + sex + session + DistanceAgr) sig(session*sex)	803.2589	18	1642.518	3.777855	1.260132 e‐01	0.9592465
3	*D*(session + habitatclass) *p*(*t* + sex + session + habitatclass) sig(session*sex)	803.8769	19	1645.754	7.013775	2.498876 e‐02	0.9842353
4	*D*(session) *p*(*t* + sex + session + DistanceAgr) sig(session*sex)	808.6710	15	1647.342	8.602042	1.129424 e‐02	0.9955295
5	*D*(session + habitatclass) *p*(*t* + sex + session) sig(session*sex)	808.0432	17	1650.086	11.346451	2.863625 e‐03	0.9983932
6	*D*(session + habitatclass) *p*(*t* + sex + session + DistanceWater) sig(session*sex)	808.0297	18	1652.059	13.319384	1.067823 e‐03	0.9994610
7	*D*(session + habitatclass) *p*(*t* + sex + session + DistanceArtificial) sig(session*sex)	808.7526	18	1653.505	14.765136	5.182725 e‐04	0.9999793
8	*D*(session) *p*(*t* + sex + session + habitatclass) sig(session*sex)	814.3411	16	1660.682	21.942217	1.432435 e‐05	0.9999936
9	*D*(session) *p*(*t* + sex + session) sig(session*sex)	817.8705	14	1663.741	25.000910	3.103759 e‐06	0.9999967

**FIGURE 2:**
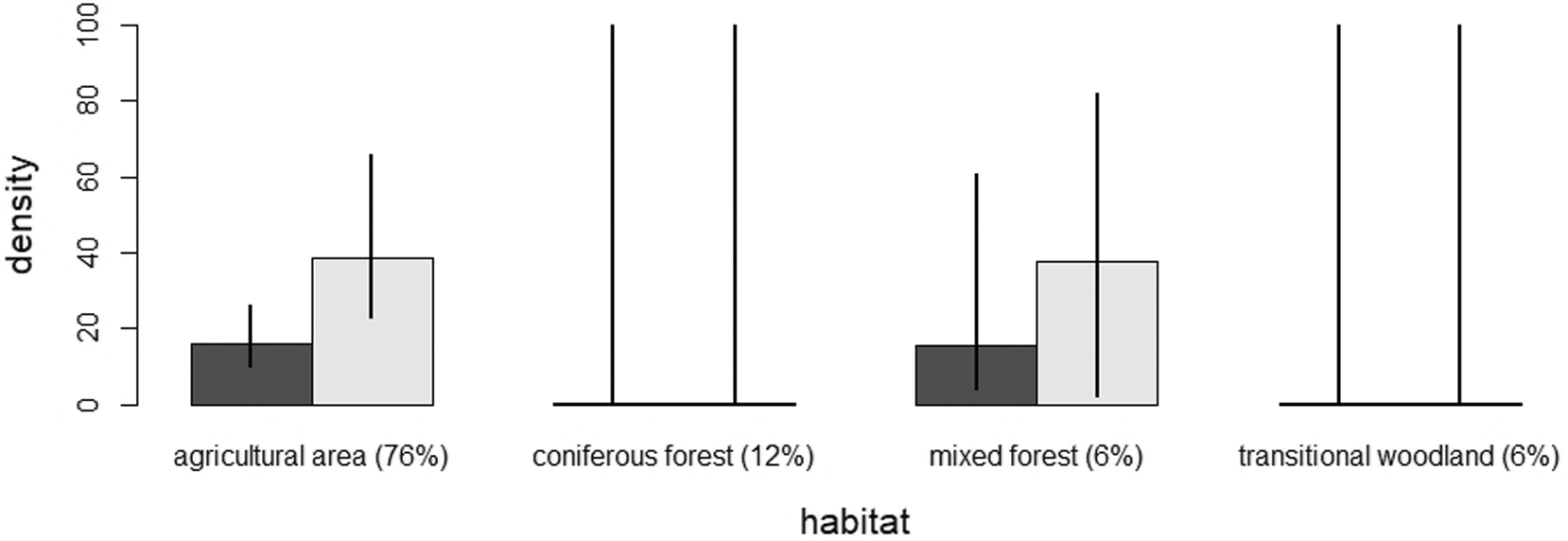
Predicted densities of white‐tailed deer per km^2^ in different landcover types in study area in southwestern Finland during 2016 (dark gray) and 2017 (light gray). The proportion of the study area covered by each habitat class is provided in brackets. The 95% confidence intervals are drawn as lines, but upper confidence limit for coniferous forest and transitional woodlands much exceed the maximum value of the *Y* axis plotted (see Table 3).

**TABLE 3:** fDNA‐SCR inferred detection probability (*p*), space use (*σ*), density (*D*) and sex ratio (*ψ*) for white‐tailed deer individuals in a heterogenous landscape during different sessions (study years) 2016 and 2017 in Southwest Finland. Estimates are provided for the most parsimonious model (m1 in Table 2). In brackets after each parameter is the unit and the scale used for model inferences (logit or exponential). Model estimates and their standard error (SE) are given on the modeled scale together with a *Z*‐test with associated *p* value for whether the estimate differs from zero. For each parameter, the intercept as well as contrasts to the intercept are given. Under “data scale” the back‐transformed value is provided (based on intercept and contrast whenever relevant), where for density also the area of one pixel (0.0144 km^2^) was taken into account such that the data‐scale estimates for density are in individuals/km^2^. Significance (*p* < .05) is indicated by bolding. Baseline detection probability (*p*_0_) refers to the probability to detect a white‐tailed deer per sample occasion at its activity center and detection probability was assumed to decline with distance from the activity center following a half‐normal function described by the space use parameter *σ*. Fecal samples were collected during each year during separate occasions (occ). Effect of habitat on detection was modeled as “distance to the nearest agricultural field” in units of meters (m) and whether the sample plot was in different habitat classes. The intercept for detection hence denotes the detection probability in session 1 (2016) for a female during the first occasion in coniferous forest at average nearest distance from an agricultural field. The intercept for sigma was for a female (f) in 2016 with contrast provided for 2017 and for males (m). The intercept for density is the habitat agricultural field in session 1 (2016). The sex ratio is the probability of an individual in the study population being a female. The model estimates for density are plotted in Figure 2.

Parameter/covariate	Estimate	SE	*z*	*p*	Data‐scale
*Detection p0 (probability to detect an individual at its activity center, logit)*					
**Intercept (*p***_**0**_ **)**	**−2.08**	**0.30**	**−6.99**		**0.11**
Male vs. female	−0.28	0.37	−0.75	.45	0.09
**Session 2 (2017)**	**−1.13**	**0.33**	**−3.39**	**.00**	**0.04**
Occ. 2	0.35	0.20	1.72	.09	0.15
Occ. 3	−0.32	0.36	−0.91	.36	0.08
**Occ. 4**	**0.84**	**0.27**	**3.16**	**.00**	**0.22**
**Occ. 5**	**0.68**	**0.27**	**2.48**	**.01**	**0.20**
**Distance to field**	**−0.01**	**0.00**	**−2.97**	**.00**	
**MixedForests**	**0.39**	**0.19**	**2.07**	**.04**	**0.15**
**Transitional wood**	**0.56**	**0.21**	**2.65**	**.01**	**0.18**
*Space use σ (meters, exponential)*					
**Intercept (f)**	**5.80**	**0.12**			**328.65**
**f 2017**	**−0.34**	**0.15**	**−2.30**	**.02**	**234.86**
**m 2016**	**−0.54**	**0.19**	**−2.86**	**.00**	**191.33**
**m 2017**	**−0.58**	**0.20**	**−2.96**	**.00**	**184.38**
*Density (individuals/pixel, exponential)*					
**Intercept**	**−1.47**	**0.25**	**−5.93**		**16.03**
**Session 2 (2017)**	**0.88**	**0.27**	**3.26**	**.00**	**38.77**
Coniferous forests	−5.55	16.36	−0.34	.73	0.06
MixedForests	−0.03	0.69	−0.04	.97	15.59
Transitional wood	−4.38	5.71	−0.77	.44	0.20
*Sex ratio ψ (probability to be female, logit)*					
Intercept	0.10	0.29	0.34	.73	0.52

Although the SCR model showed density to differ across habitat classes, the uncertainty around some of the estimates was large (Figure 2; Table 3). Point estimates of the model suggest that white‐tailed deer densities were particularly high in agricultural areas and mixed forest, but low in coniferous forests and transitional woodlands during both years (Figure 2). Density was clearly higher during the second year compared with the first year (Figure 2; Table 3). Detection probability was highest in transitional woodlands, second highest in mixed forests and lowest in coniferous forests. Detection probability decreased with distance to agricultural areas. During the first year, detection probability was higher than during the second year (Table 3).

The space use parameter *σ* differed between the sexes, sampling year and their interaction. Female *σ* was lower in the second sampling year compared with the first; female *σ* was higher than that of males (Table 3). For males, distances moved between consecutive recaptures were on average 233 m (95% CI: 59–655), and for females 295 m (0–1081). The sex ratio (*ψ*), based on SCR under the top model, was about equal (0.52).

Overall, white‐tailed deer density was estimated to be higher when landscape covariates were included compared with assuming a homogeneous landscape. The homogeneous top model (model 1 in Table A2) predicted that the overall density of white‐tailed deer across the whole state space was 11.2 (11.0–11.4) white‐tailed deer/km^2^ in 2016 and 25.5 (25.0–26.0) white‐tailed deer/km^2^ in 2017. The heterogeneous top model with habitat covariates (m1 in Table 2) predicted that the overall density was 13.1 (12.6–13.6) white‐tailed deer/km^2^ in 2016 and 31.7 (30.5–32.9) white‐tailed deer/km^2^ in 2017.

## 4 DISCUSSION

We found that inclusion of habitat heterogeneity was important when estimating density and detection of white‐tailed deer: Including landscape heterogeneity significantly increased prediction performance of the SCR models. While we found support that white‐tailed deer density varied across habitat classes, indicating second‐order habitat selection, the uncertainty around the point estimates for density in these habitat classes was large. Based on point estimates, however, our findings indicate that white‐tailed deer densities were particularly high in agricultural areas and mixed forests but low in coniferous forests and transitional woodlands. Including landscape heterogeneity furthermore increased the overall density estimate when compared with density estimated under the best supported homogeneous (constant density without habitat covariates) SCR model. The higher density estimated by the heterogeneous model may be a result of the greatest proportion of the state space consisting of agricultural areas (76%), which is also the preferred habitat (together with mixed forests which are, however, relatively uncommon). Our findings were intuitive as fields in the study area were predominantly crop fields, and these present habitat with good food resources for the white‐tailed deer during the autumn months this study was conducted.

One of the advantages of the SCR approach used here is that densities can be inferred for habitat where sampling is not possible; in our case sample plots could not be placed in agricultural fields due to legal restrictions on access. Nevertheless, uncertainty around estimates of habitat‐specific density was large, especially for the habitat classes where density was inferred to be low. Thus, while the heterogeneous SCR model finds clear evidence for a density‐habitat relationship (i.e. habitat selection), it remains challenging to provide habitat‐specific density estimates. Heterogeneous SCR models are data hungry, so sampling over a longer time period (e.g. 6 weeks) while retaining short sampling intervals could provide more power for inferring densities. Longer time periods would require careful consideration of population closure and stability of habitat (especially the agricultural fields). Nevertheless, a strength of the SCR approach is that it estimates habitat‐specific densities (second order habitat selection), while accounting for third order habitat selection through the effect of habitat on detection probability. Here we find that the detection probability of white‐tailed deer was highest in transitional woodlands followed by mixed forest and was lowest in coniferous forest. Detection probability also was lower with increasing distance to agricultural fields. Thus, space was not used equally; white‐tailed deer preferred to be close to fields and in transitional woodlands compared with coniferous forest. In our study area, transitional woodlands were typically clear‐cuts and white‐tailed deer may use those areas there due to food resources such as shrubs and young seedlings. Similarly, mixed forests, which include deciduous trees and typically have an undergrowth of small shrubs likely offer more nutritious food compared with coniferous forests.

Our study was conducted during 2–3 weeks in late summer. Clearly, habitat preferences later in the year likely differ from these late‐summer estimates, especially during winter when there are no seed crops and snow covers the agricultural fields. In general, snow cover affects the availability of food and movement of individuals (Andersson & Koivisto, 1980). In winter, instead of fields and other open areas, white‐tailed deer may favor moving more in mature forests including coniferous forests where snow depth is less allowing easier movement and access to food. Also, in winter, white‐tailed deer typically eat more conifers as leaves of deciduous trees are not available (Kankaanpää, 1989; Martinka, 1968; Weckerly & Nelson, 1990). Thus, habitat‐density relationships likely vary dramatically throughout the year. The white‐tailed deer was introduced in Finland about 90 years ago and has increased dramatically in numbers in the last decade (Aikio & Pusenius, 2022). Improving inference of white‐tailed deer density, e.g. by better understanding density‐habitat relationships, is therefore important in developing targeted management of this species in Finland.

Density estimates in this study were high compared with regional pre‐harvest estimates of the area, which were 3.9 and 4.2 individuals per km^2^ in 2016 and 2017, respectively (Brommer et al., 2021). Our pre‐harvest estimates were 11 and 31 individuals/km^2^, respectively. Comparing our estimates to regional estimates is complicated by the fact that regional estimates are based on multiple sources of information including time‐series of hunting statistics and describe the average density over a large area, whereas our estimates are based on a snap‐shot (2–3 weeks sampling) conducted in a small (approximately 3 km^2^) area. It is nevertheless noteworthy that our findings underline that white‐tailed deer density can locally be up to almost an order of magnitude higher than the regional average. Clearly, understanding why white‐tailed deer aggregate in some localities (and presumably not in others) is a major future challenge.

We found an approximately three‐fold increase in density between the two study years; a change that is already apparent in the fact that we recorded more individuals (and also recaptured more individuals) in 2017 compared with 2016. Importantly, however, the increase in white‐tailed deer density that we recorded does not reflect population growth, because the spatial scale of our study area is limited. Our fecal DNA sample plots covered a relatively small forest patch which is surrounded by fields. White‐tailed deer likely aggregated in this forest patch offering cover while surrounded by good food resources from fields, potentially more so in 2017 compared with 2016. Furthermore, in 2017, the fecal collection was conducted 1 month earlier than in 2016 (due to a change in the starting date of hunting), which also may have affected the difference in density estimates between years, e.g. due to better availability of food including the crops on the field, as well as behavioral differences. In particular, white‐tailed deer males form so‐called bachelor groups of multiple deer from late winter through summer (Hawkings & Klimstra, 1970; Sorensen & Taylor, 1995). In August, males still move more in bachelor groups of multiple males than in September, which was noticed by wildlife cameras that were simultaneously recording for another study (Brommer et al., 2021). To conclude, various factors may underlie changes in the density and sex ratios in the study area between the 2 years studied. Further study, especially covering areas with different habitats and different periods of the year, and incorporating changes in habitat (e.g. harvest of crop) are needed to generalize our findings and improve our knowledge of factors affecting local white‐tailed deer densities.

One powerful aspect of the SCR approach is that potentially a large fraction of a population can be identified and followed, thereby allowing population‐level insights (Royle et al., 2018). In our case, more than 100 individuals were identified and about 50% of them were recaptured using non‐invasive fecal DNA. Nevertheless, only 32% of fecal samples allowed establishing individuals identities. This is low compared with DNA‐based individual identification of white‐tailed deer in the U.S.A. (66%; Goode et al., 2014). One reason is that DNA in feces is degraded, hampering amplification, and individual identification requires successful amplification of more microsatellites (11) in Finland compared with United States (7; Goode et al., 2014). This is because the introduction of the white‐tailed deer in Finland was based on only few individuals and the population therefore has a reduced allelic richness (Kekkonen et al., 2012). A methodological challenge in using fecal DNA‐based SCR for deer and other animals that often live in groups is that activity centers may show some level of non‐independence across individuals. Although density inference should not be biased (Bischof et al., 2020; Russell et al., 2012), a generally applicable way to accommodate dependencies of home ranges would improve inferences (Bischof et al., 2020). Although the approaches may vary between studies, our findings add to the growing SCR‐based evidence that it is important to consider the spatial heterogeneity of an area and habitat selection of animals. By acknowledging that landscapes are heterogeneous and incorporating that into our analyses, we quantify habitat selection, which captures crucial ecology in terms of how the organism uses its environment both in terms of population density and in terms of individual space use.

In this study, we provide insight into habitat selection of white‐tailed deer in Finland, which thus far has not been examined in Finnish white‐tailed deer populations. Our findings point to the importance of considering habitat structure in density estimation in wildlife management. Information on habitat preference can be used as a covariate when estimating densities over different landscapes for setting management goals. With this information, hunting licenses can be allocated in areas with more favorable habitats in order to reduce damage (i.e. to forestry or agriculture) caused by dense white‐tailed deer populations. Better knowledge of where and when during the year white‐tailed deer densities are high may also help to minimize deer‐vehicle collisions. Finally, research, like this study, that provides information on habitat selection of white‐tailed deer in Finland offers new insights into little‐studied basic ecological aspects of a species with a remarkable capacity to thrive in different environments.

## AUTHOR CONTRIBUTIONS

**Jenni Poutanen:** Conceptualization (equal); data curation (lead); formal analysis (lead); funding acquisition (equal); investigation (lead); writing – original draft (lead); writing – review and editing (equal). **Angela K. Fuller:** Formal analysis (equal); writing – review and editing (equal). **Jyrki Pusenius:** Funding acquisition (equal); project administration (equal); writing – review and editing (equal). **J. Andrew Royle:** Formal analysis (equal); writing – review and editing (equal). **Mikael Wikström:** Funding acquisition (equal); project administration (equal); writing – review and editing (equal). **Jon E. Brommer:** Conceptualization (equal); data curation (equal); formal analysis (equal); funding acquisition (equal); project administration (lead); supervision (lead); writing – original draft (equal); writing – review and editing (equal).

## CONFLICT OF INTEREST

The authors declare they have no conflicting interests.

## Data Availability

The fecal DNA data (coordinates of the sampling plots, microsatellite genotypes of the individuals and individual encounter histories) for spatial capture‐recapture modeling and the R script for conducting these models will be available from the repository Dryad upon acceptance (https://doi.org/10.5061/dryad.v15dv420s).

## APPENDIX 1

Density‐habitat relationships of white‐tailed deer (Odocoileus virginianus) in Finland" by Poutanen et al.

**TABLE A1:** Specifics of the 14 microsatellite loci used in this study on white‐tailed deer in Finland in two study years 2016 and 2017. We report basic statistics on their allele richness, heterozygosity and deviation from Hardy‐Weinberg equilibrium (HWE).

Locus	No. of alleles	Allele range (bp)	Expected heterozygosity (*H*_*e*_)	Observed heterozygosity (*H*_*o*_)	HWE
2016					
BM4107	5	139–166	0.56	0.55	0.4321
Cervid1	6	173–187	0.77	0.76	0.3855
Rt5	7	154–172	0.79	0.79	0.7377
OCAM	4	206–212	0.70	0.58	0.4959
INRA011	4	193–206	0.55	0.61	0.9591
BM203	8	210–233	0.70	0.61	0.2280
ETH152	7	176–197	0.76	0.68	0.1061
OarFCB193	6	97–123	0.74	0.71	0.9232
BL25	4	177–182	0.60	0.61	0.8488
K	2	198–202	0.49	0.55	0.4953
O	6	188–218	0.58	0.61	0.8233
BM6506	4	195–204	0.64	0.53	0.1485
*D*	6	159–187	0.76	0.87	0.2654
*p*	5	214–242	.75	.63	.5486
Mean	5.3	–	0.67	0.65	–
2017					
BM4107	6	139–166	0.62	0.62	0.5647
Cervid1	6	173–187	0.76	0.76	0.7272
Rt5	7	154–172	0.78	0.73	0.6841
OCAM	5	206–216	0.75	0.58	0.4139
INRA011	4	193–206	0.66	0.64	0.7602
BM203	9	210–233	0.70	0.56	0.0089^a^
ETH152	9	174–197	0.78	0.79	0.2722
OarFCB193	6	97–123	0.76	0.68	0.5004
BL25	5	177–192	0.57	0.52	0.7886
K	3	198–206	0.51	0.62	0.0332^a^
O	6	188–218	0.46	0.44	0.9949
BM6506	5	195–204	0.69	0.70	0.9386
*D*	6	159–187	0.76	0.79	0.1473
*p*	7	214–242	.74	.56	.1745
Mean	6	–	0.62	0.62	–

^a^ Deviations from Hardy‐Weinberg equilibrium.

**TABLE A2:** SCR models assuming a homogeneous landscape when estimating white‐tailed deer population in southwestern Finland during 2016–2017. The SCR models density (*D*), detection probability *p*_0_ and space use sigma (*σ*) as a function of covariates, which here are session (year), occasion (*t*), and sex (sex). For each parameter (*D*, *p* and sig), the covariates included are included between brackets either as additive terms (+) or interacting (*). For each model, the log‐likelihood (LogL), number of parameters (*K*), Akaike Information Criterion (AIC) are provided. Models are ranked in terms of AIC, and the difference in AIC between each model and the most parsimonious model, model 1 (ΔAIC) is provided as well as the AIC weight of the model and cumulative (summed) weight of each model together with the models ranked above it.

	Model	logL	*K*	AIC	ΔAIC	AIC weight	Cumulative weight
1	*D*(session) *p*(*t* + sex + session) sig(session*sex)	817.8683	14	1663.737	0	0.372351	0.372351
2	*D*(session) *p*(*t* + sex + session) sig(session)	820.1075	12	1664.215	0.478442	0.29313	0.665481
3	*D*(session) *p*(*t**session + sex) sig(session)	820.1075	12	1664.215	0.478442	0.29313	0.958611
4	*D*(session) *p*(*t* + sex) sig(session)	824.4422	11	1670.884	7.147799	0.010443	0.969054
5	*D*(session) *p*(*t* + session) sig(session)	824.6474	11	1671.295	7.558151	0.008506	0.97756
6	*D*(session) *p*(*t**session) sig(session)	824.6474	11	1671.295	7.558151	0.008506	0.986066
7	*D*(.) *p*(*t* + session) sig(sex + session)	825.3815	11	1672.763	9.026306	0.004082	0.990148
8	*D*(.) *p*(*t* + sex + session) sig(session + sex)	824.8797	12	1673.759	10.0228	0.00248	0.992629
9	*D*(.) *p*(*t* + sex + session) sig(.)	827.0322	10	1674.064	10.32785	0.00213	0.994758
10	*D*(session) *p*(session*sex) sig(session)	828.0616	9	1674.123	10.38656	0.002068	0.996826
11	*D*(.) *p*(*t* + sex + session) sig(session)	826.3990	11	1674.798	11.06136	0.001476	0.998302
12	*D*(session) *p*(session + sex) sig(session + sex)	829.0787	9	1676.157	12.42084	0.000748	0.99905
13	*D*(.) *p*(*t* + sex) sig(sex + session)	828.4259	11	1678.852	15.11525	0.000194	0.999244
14	*D*(.) *p*(*t*) sig(session + sex)	829.5957	10	1679.191	15.45483	0.000164	0.999408
15	*D*(.) *p*(*t*) sig(session + sex)	829.5957	10	1679.191	15.45483	0.000164	0.999572
16	*D*(session) *p*(*t*) sig(session)	829.6167	10	1679.233	15.49675	0.000161	0.999733
17	*D*(session) *p*(session + sex) sig(.)	833.2317	7	1680.463	16.72681	8.69E‐05	0.99982
18	*D*(.) *p*(session + *t*) sig(.)	831.5100	9	1681.02	17.28345	6.58E‐05	0.999886
19	*D*(.) *p*(session + *t*) sig(session)	831.0918	10	1682.184	18.44693	3.67E‐05	0.999922
20	*D*(session) *p*(.) sig(session + sex)	834.2520	7	1682.504	18.76727	3.13E‐05	0.999954
21	*D*(session) *p*(session) sig(session)	834.8514	7	1683.703	19.96611	1.72E‐05	0.999971
22	*D*(.) *p*(session + sex) sig(sex)	835.3587	7	1684.717	20.9808	1.04E‐05	0.999981
23	*D*(.) *p*(session + sex) sig(.)	837.2110	6	1686.422	22.68537	4.41E‐06	0.999986
24	*D*(session) *p*(session) sig(.)	837.4305	6	1686.861	23.12446	3.54E‐06	0.999989
25	*D*(.) *p*(*t*) sig(session)	834.6859	9	1687.372	23.63512	2.75E‐06	0.999992
26	*D*(.) *p*(*t*) sig(sex)	834.9153	9	1687.831	24.09398	2.18E‐06	0.999994
27	*D*(.) *p*(*t*) sig(sex)	834.9153	9	1687.831	24.09398	2.18E‐06	0.999996
28	*D*(.) *p*(*t* + sex) sig(sex)	834.1021	10	1688.204	24.46766	1.81E‐06	0.999998
29	*D*(.) *p*(*t* + sex) sig(.)	835.6012	9	1689.202	25.46573	1.10E‐06	0.999999
30	*D*(session) *p*(*t* + sex) sig(.)	835.4936	10	1690.987	27.25052	4.50E‐07	1
31	*D*(.) *p*(session) sig(.)	841.6896	5	1693.379	29.6425	1.36E‐07	1
32	*D*(.) *p*(session) sig(session)	841.2827	6	1694.565	30.82882	7.53E‐08	1
33	*D*(.) *p*(*t*) sig(.)	840.7396	8	1697.479	33.7426	1.75E‐08	1
34	*D*(.) *p*(.) sig(session)	843.8428	5	1697.686	33.94895	1.58E‐08	1
35	*D*(.) *p*(.) sig(sex)	843.9382	5	1697.876	34.13981	1.44E‐08	1
36	*D*(.) *p*(sex) sig(sex)	843.0880	6	1698.176	34.43938	1.24E‐08	1
37	*D*(.) *p*(sex) sig(.)	844.5648	5	1699.13	35.39294	7.68E‐09	1
38	*D*(session) *p*(*t*) sig(.)	840.5904	9	1699.181	35.44424	7.49E‐09	1
39	*D*(session) *p*(.) sig(sex)	843.8684	6	1699.737	36.00017	5.67E‐09	1
40	*D*(session) *p*(sex) sig(.)	844.5049	6	1701.01	37.27324	3.00E‐09	1
41	*D*(.) *p*(.) sig(.)	849.7511	4	1707.502	43.76559	1.17E‐10	1
42	*D*(session) *p*(.) sig(.)	849.6673	5	1709.335	45.59795	4.67E‐11	1
